# The bat community of Haiti and evidence for its long-term persistence at high elevations

**DOI:** 10.1371/journal.pone.0178066

**Published:** 2017-06-02

**Authors:** J. Angel Soto-Centeno, Nancy B. Simmons, David W. Steadman

**Affiliations:** 1 Department of Mammalogy, American Museum of Natural History, New York, NY, United States of America; 2 Department of Ornithology, Florida Museum of Natural History, Gainesville, FL, United States of America; Brown University, UNITED STATES

## Abstract

Accurate accounts of both living and fossil mammal communities are critical for creating biodiversity inventories and understanding patterns of changing species diversity through time. We combined data from from14 new fossil localities with literature accounts and museum records to document the bat biodiversity of Haiti through time. We also report an assemblage of late-Holocene (1600–600 Cal BP) bat fossils from a montane cave (Trouing Jean Paul, ~1825m) in southern Haiti. The nearly 3000 chiropteran fossils from Trouing Jean Paul represent 15 species of bats including nine species endemic to the Caribbean islands. The fossil bat assemblage from Trouing Jean Paul is dominated by species still found on Hispaniola (15 of 15 species), much as with the fossil bird assemblage from the same locality (22 of 23 species). Thus, both groups of volant vertebrates demonstrate long-term resilience, at least at high elevations, to the past 16 centuries of human presence on the island.

## Introduction

Caribbean islands (the West Indies) are among the top ten biodiversity hotspots and currently sustain some of the highest levels of endemism in the world [1]. These islands have sustained massive losses of species in many mammalian taxa since the last glacial maximum (~25–18ka). For example, the Caribbean islands once hosted a terrestrial, non-volant mammal diversity of ±95 species including insectivores, primates, rodents, and sloths, but at least ~80% of these became extinct in the late Quaternary [2,3]. Many of these losses took place during the mid- to late Holocene, a time interval characterized by climates, sea levels, and island areas similar to those of today [2–6].

Unlike in the nearby Bahamas, the island of Hispaniola (Dominican Republic + Haiti) remained largely unaffected in physical geography by climate driven sea-level changes during the Pleistocene-Holocene transition (PHT, centered around 15–9 ka) because of its high topographic profile and deep surrounding seas [7]. It is estimated that land area loss resulting from sea-level rise from the Last Glacial Maximum to the present in Hispaniola was as little as 6% [7]. Thus, Hispaniola is an ideal place to study the loss vs. long-term persistence of species without the need to consider changing island area or the loss of available terrestrial habitats. In this context, paleontological studies can help us understand the patterns, causes, and timing of faunal change, thereby providing a long-term perspective useful to improve our knowledge of current biodiversity. Documentation of fossil diversity and its comparison with modern biotas can help assess how abiotic (e.g., climate) and biotic (e.g., anthropogenic disturbance) historical events affected these insular communities.

Bats are the most species-rich native mammals in the Caribbean. The bat fauna of the Caribbean once featured at least 67 species, of which 12 (18%) became extinct sometime during the late Quaternary [2,8]. The island of Hispaniola today supports 18 (32%) of the 56 extant species of Caribbean bats [9–11]. The Hispaniolan fossil bat fauna has been studied in some detail from 10+ fossil sites, including two recently discovered flooded sinkholes in the Dominican Republic [12–15]. The bat fauna of adjacent Haiti has received less attention. Klingener et al. [16] compiled a comprehensive dataset of extant bats encompassing 17 localities surveyed between 1973 and 1975 along the Tiburon Peninsula in southern Haiti. While other authors have studied the Haitian fossil bat fauna from various localities [12,13,17–19], documentation of the fossil bat fauna of Haiti is scant and lacks a comparison with modern inventories. Furthermore, the chronological control on these fossil assemblages has been poorly resolved beyond being “late Quaternary.”

The combination of extant and fossil species inventories with a chronological framework for a paleontological site can yield crucial data on such interrelated topics as species turnover, population changes, range shifts, and species and community composition. For example, a radiocarbon dated set of fossil bats from Abaco (The Bahamas) demonstrated that this island lost five (50%) of its species within the past four millennia [8]. In this paper, we present a comprehensive literature review of the bats of Haiti, including all previous records of living and fossil species. Our compilation builds on that of Velazco et al. [15] for bats from the Dominican Republic. We also report bat fossils from 14 Haitian localities not previously documented, including a detailed description and chronology for the fossil bats of Trouing Jean Paul, a rich deposit in a limestone sinkhole in the mountains of southeastern Haiti.

## Materials and methods

### Literature and museum records

To develop a complete list of the documented extant and fossil bat species from Haiti, we performed three independent literature searches during August 2016. The first consisted of an Internet search in Web of Science across all years using the key words “Haiti” and “Hispaniola” within the title (TI) or as a topic (TS), and refined by “Mammalia.” A second Internet search was performed in Google Scholar using the key words “Haiti OR Hispaniola” and “bat OR Chiroptera.” Sources including extant or fossil species inventories and biogeographic or species accounts within the geographic scope of the Caribbean were considered relevant in our search, which resulted in eight articles fitting these criteria (Table 1). The third search consisted of a more classical approach scanning the references included in the literature cited of each of the relevant articles.

**Table 1:** Bat diversity of Hispaniola (Dominican Republic + Haiti).

Family	All bat species reported in Hispaniola	Extant Hispaniola^a^[15,16,20]	Extant Parc National La Visite[21]	Trouing Jean Paul^b^	Diquini^c^[14,18]	Gonâve Island^c^[13,19]	Port-de-Paix^c^[17]	Saint-Michel-de-l’Atalaye^c^[14,18]	La Selle^c^^ˆ^[17]
Molossidae	*Molossus molossus*	x							
	*Nyctinomops macrotis*	x		x					
	*Tadarida brasiliensis*	x	x	x				x	
	[*Tadarida* sp.]								x
Mormoopidae	*Mormoops blainvillei*	x				x		x	
	*Mormoops magna*^†^								
	*Mormoops megalophylla*^*#*^								
	*Pteronotus macleayii*	?							
	*Pteronotus parnellii*	x		x	x	x			
	*Pteronotus quadridens*	x		x					
	*Pteronotus* sp.^†^								
Natalidae	*Chilonatalus micropus*	x		x					
	*Natalus major*	x		x					
Noctilionidae	*Noctilio leporinus*	x							
Phyllostomidae	*Brachyphylla nana*	x		x			x	x	
	*Erophylla bombifrons*	x		x			x	x	
	*Monophyllus redmani*	x	x	x	x	x			
	*Phyllonycteris poeyi*	x		x	x			x	
	*Macrotus waterhousii*	x		x	x	x		x	
	*Artibeus jamaicensis*	x			x	x	x	x	
	*Phyllops falcatus* (*= haitiensis*^‡^)	x	x	x	x		x	x	
Vespertilionidae	*Eptesicus fuscus*	x	x	x				x	x
	*Lasiurus cinereus*	?		x					
	Lasiurus intermedius^*^								
	*Lasiurus minor*	x		x					

^a^ Species reported in VertNet (accessed 1 December 2016) and reported in the literature

^b^ fossil bats reported by this study

^c^ fossil reported in the literature.

Extinct species = †

species extirpated in the Caribbean = #

species extirpated in Hispaniola = *

specific epithet synonym in previous publications = ‡

species of unknown status =?.

Taxa in brackets not necessarily different from those identified more precisely.

We also searched for all museum specimens of bats in VertNet (www.vertnet.org; accessed on 1 December 2016) to complement our extant species list. This search included specimen data from 12 institutions (American Museum of Natural History, AMNH; University of Kansas Biodiversity Institute, KU; Natural History Museum of Los Angeles County, LACM; Museum of Comparative Zoology, MCZ; Muséum d'histoire naturelle de la Ville de Genève, MHNG; UC Berkeley Museum of Vertebrate Zoology, MVZ; Natural History Museum, NHMUK; Museum of Texas Tech University, TTU; Florida Museum of Natural History, UF; University of Michigan Museum of Zoology, UMMZ; National Museum of Natural History Smithsonian Institution, USNM; and Yale Peabody Museum, YPM).

### Trouing Jean Paul cave site

**Fig 1:**
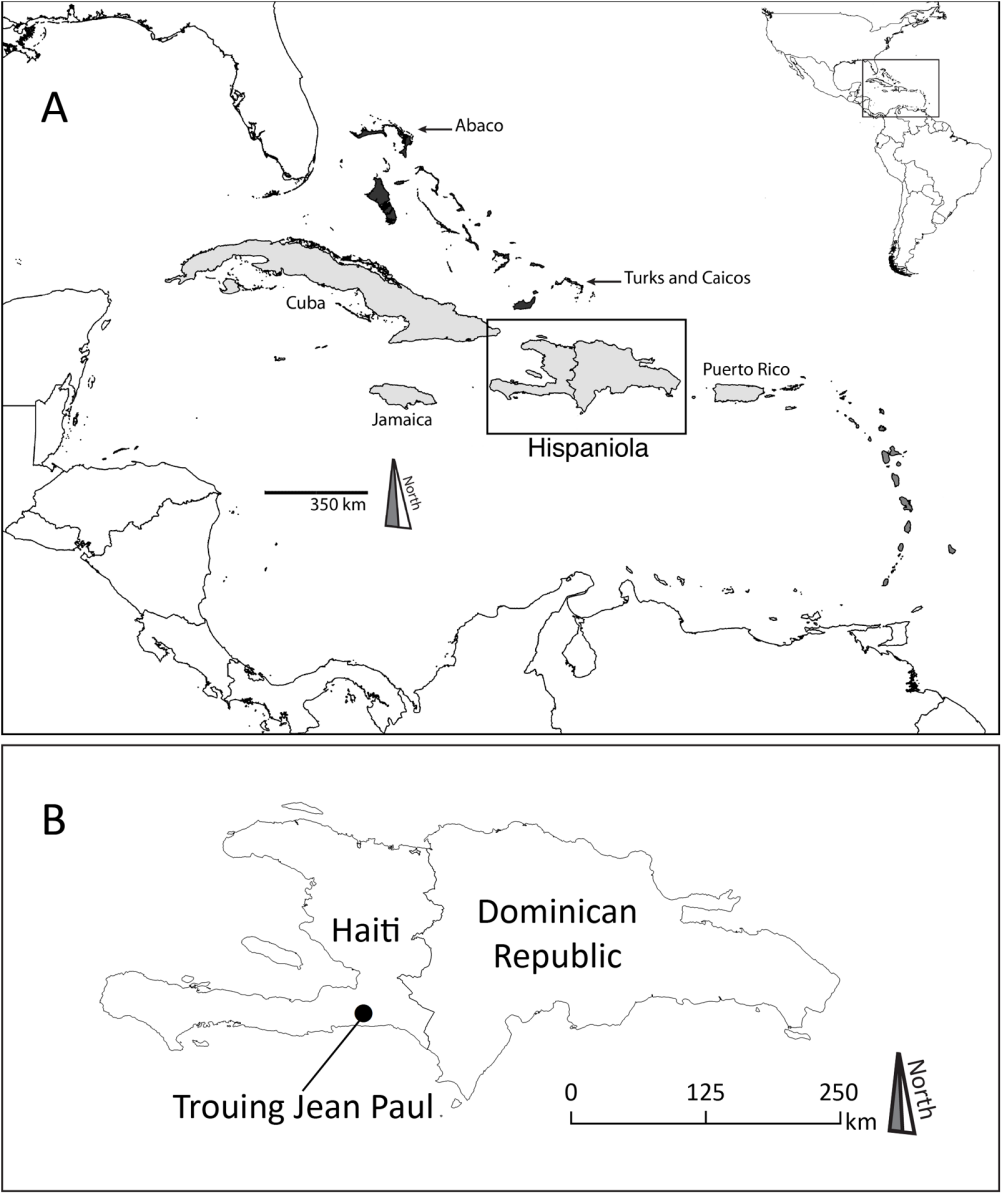
**Map of the Caribbean (A).** Major island groups highlighted as: Greater Antilles = light gray, Lesser Antilles = dark gray, and The Bahamas = black. The inset indicates the island of Hispaniola as reference including the locality of Trouing Jean Paul (B).

**Fig 2:**
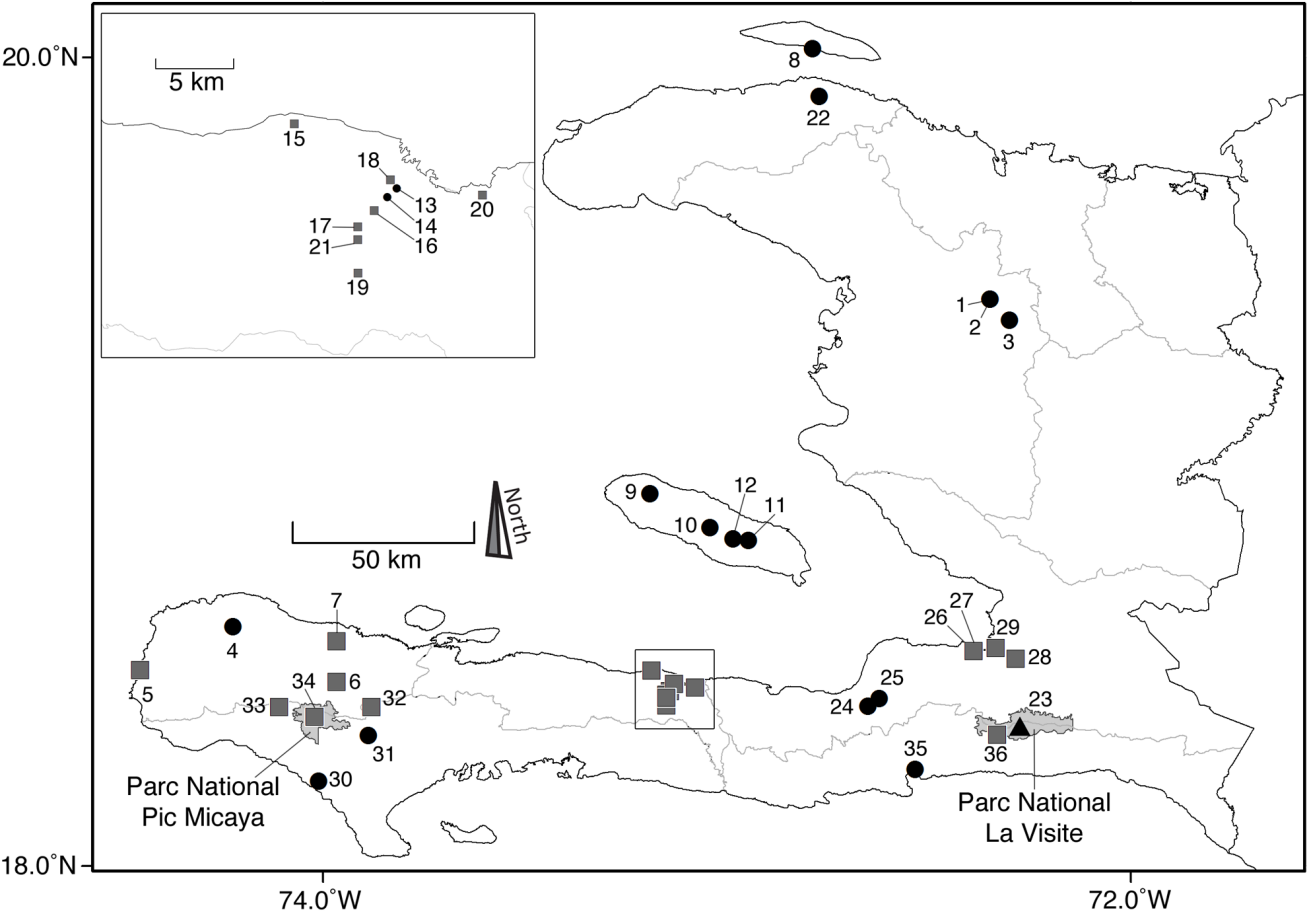
Map of Haiti indicating localities of living bat species (squares) and fossil bat species (circles). Boundaries of Parc National Pic Macaya and Parc National La Visite are indicated with light gray polygons. The locality of Trouing Jean Paul is indicated by a triangle (23) within Parc Nacional La Visite. Inset map shows detail of localities in the Province of Nippes. Numbers refer to the specific localities listed in S1 Table.

Trouing Jean Paul (TJP) is a limestone sinkhole cave located ~22 km south of Port-au-Prince in Parc National La Visite, Massif de la Selle, Haiti (18.33˚N, -72.28˚W; Figs 1 and 2). TJP is a high elevation site (~1825 m) that was discovered in 1983 and excavated primarily in February 1984 by a field team led by Charles A. Woods. Relevant permits and documentation associated with the fossil excavations were obtained by C. A. Woods at the Florida Museum of Natural History, University of Florida (UF) from 1978 to 1984. These fossils were loaned to us for study by the UF Division of Vertebrate Paleontology, where permits and field notes are archived. This cave is rich in vertebrate fossils including specimens of the insectivorans *Nesophontes* sp. (Nesophontidae) and *Solenodon paradoxus* (Solenodontidae), and the rodents *Brotomys voratus* (Echimydae) and *Plagiodontia aedium* (Capromyidae). Additionally, the non-passerine fossil bird community of TJP includes 23 species, only one of which (the woodcock *Scolopax brachycarpa*) is extinct [22,23].

The entrance chamber of TJP is about 20 m long at an incline of 30˚, and opens to passages that lead to five rooms. The fossils reported herein were obtained from Room 1, which is about 5 m wide by 9 m long, and divided in three main areas: Eastern, Southern, and Western Pockets. Surface fossils were collected and mapped in four-part grid coordinates, as explained in Steadman and Takano [22]. We identified all diagnostic bat fossils to species via direct comparison with modern bones (e.g. dentaries, humeri, and skulls) from the UF and AMNH collections. Reference material included all species of bats listed as resident to Hispaniola in [10,11,15,24]. We estimated the overall minimum number of individuals (MNI) for each species from the number of unique bones (e.g., left humerus) assigned to it in the fossil material. The total of the most abundant bone for each species is its MNI value. The bat fossils from TJP are housed in the UF Vertebrate Paleontology Collection in Gainesville, FL, and openly accessible through the VertNet portal (www.vertnet.org).

Detailed radiocarbon (^14^C) chronologies allow us to understand how the timing of changes in populations or species may be related to specific historical events [6,25]. From TJP, six Accelerator Mass Spectrometer (AMS) ^14^C dates were obtained from purified collagen of individual bones of the extinct woodcock (*Scolopax brachycarpa* [22,23]). These six bones, each representing a different individual woodcock, were selected from scattered locations across the surface of Room 1 in TJP, with each of these locations also having abundant identified bat fossils. Thus, the *Scolopax* AMS ^14^C dates can be used to estimate the age of the bat fossils from TJP. All AMS ^14^C dates, reported in calendrically calibrated years before present (Cal BP), represent 95% (2δ) estimates and were performed at Beta Analytic, Inc., Miami, FL. Specific details of laboratory and calibration methods can be found at www.radiocarbon.com.

## Results

A total of 24 bat species have been reported for Hispaniola including extant and fossil records (Table 1). Three species of Mormoopidae (*Mormoops magna*, *M*. *megalophylla*, and *Pteronotus* sp.) and one member of Verspertilionidae (*Lasiurus intermedius*), each known only from Dominican Republic are extinct on Hispaniola [15]. The report of *Tadarida* sp. (Molossidae) by Miller [19], based on two mandibles, most likely represents *Tadarida brasiliensis* and not a taxon new for the island. Recent published accounts of the complete list of extant bats in Hispaniola (including both the Dominican Republic and Haiti) reported 18 species for the island, [10,11,26], but we found modern records of 20 species (Table 1). The two species on our list that were not documented in previous studies are *Pteronotus macleayii* (Mormoopidae; NHMUK 1842.11.24) in Haiti and *Lasiurus cinereus* (Vespertilionidae; USNM 105704) in Dominican Republic, each represented by a single specimen.

Our study of museum collections and published records reveals that 36 localities have been sampled for bats in Haiti (Fig 2; S1 Table). Extant bats have been documented from 17 localities [16,20,21]. Fossil remains from five localities have been published [13,17–19] and 14 additional fossil localities are documented herein from the Vertebrate Paleontology Collection at UF (Table 1). Detailed information on each locality, including the 14 fossil sites, is provided in S1 Table.

An unusually rich source of data on prehistoric faunas of Haiti is the fossil assemblage from TJP, which consists of many thousands of bones of small vertebrates (bats, insectivorans, rodents, birds, frogs, lizards, and snakes). This site is a roost of the two extant species of tytonid owls on Hispaniola, which are also found as fossils at TJP [22]. *Tyto alba* (Barn Owl) is widely distributed across the Caribbean, whereas *T*. *glaucops* (Ashy-faced Owl) is endemic to Hispaniola [27,28].

**Fig 3:**
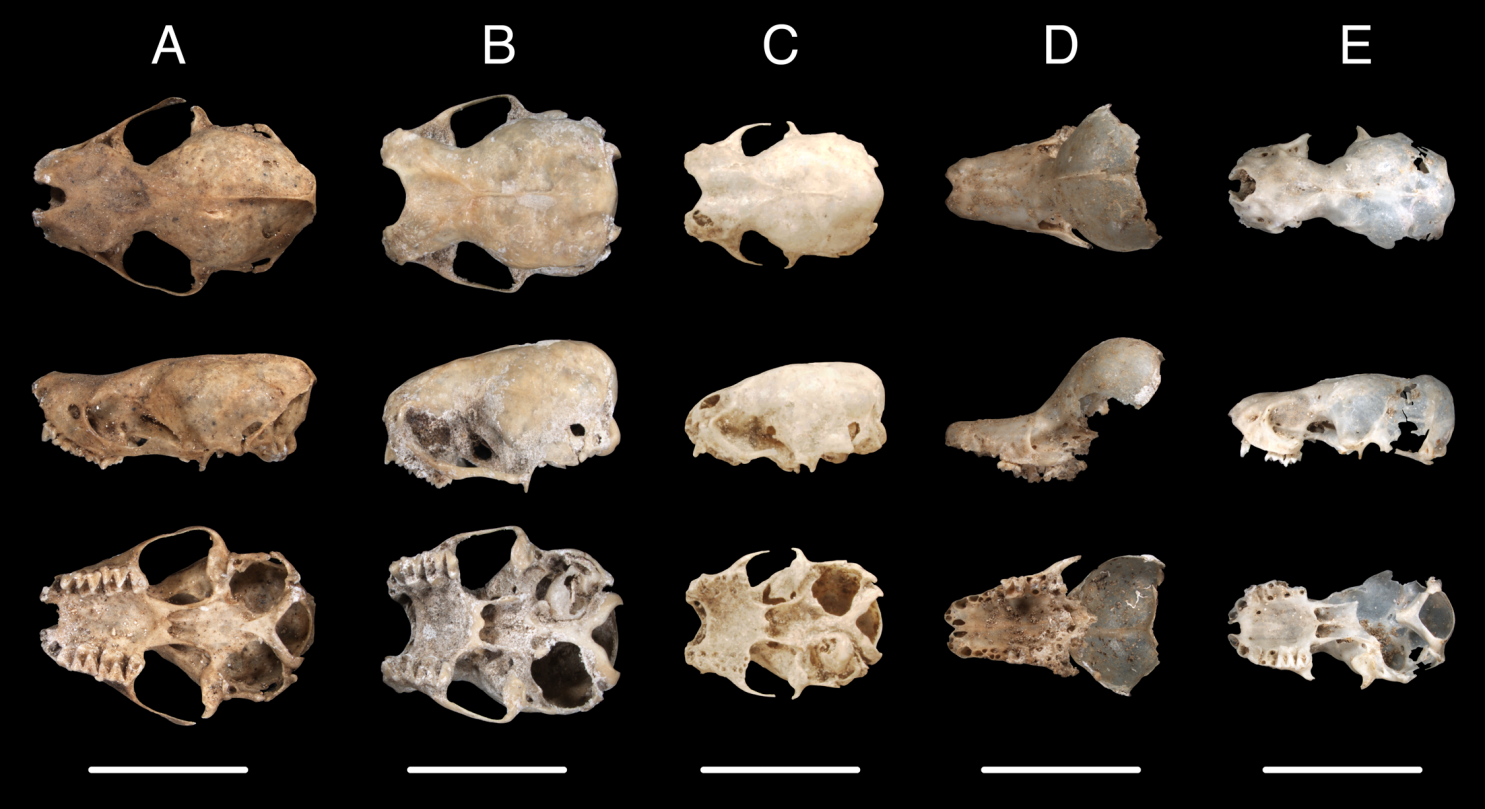
Fossil crania of bats from Trouing Jean Paul, Haiti, shown as dorsal (top), lateral (middle), and ventral (bottom) views. Left to right: Vespertilionidae—*Eptesicus fuscus* (A; UF 281797), *Lasiurus cinereus* (B; UF 282166), *Lasiurus minor* (C; UF 307426), Natalidae—*Natalus major* (D; UF 307471), and Molossidae—*Tadarida brasiliensis* (E; UF 282589).

**Fig 4:**
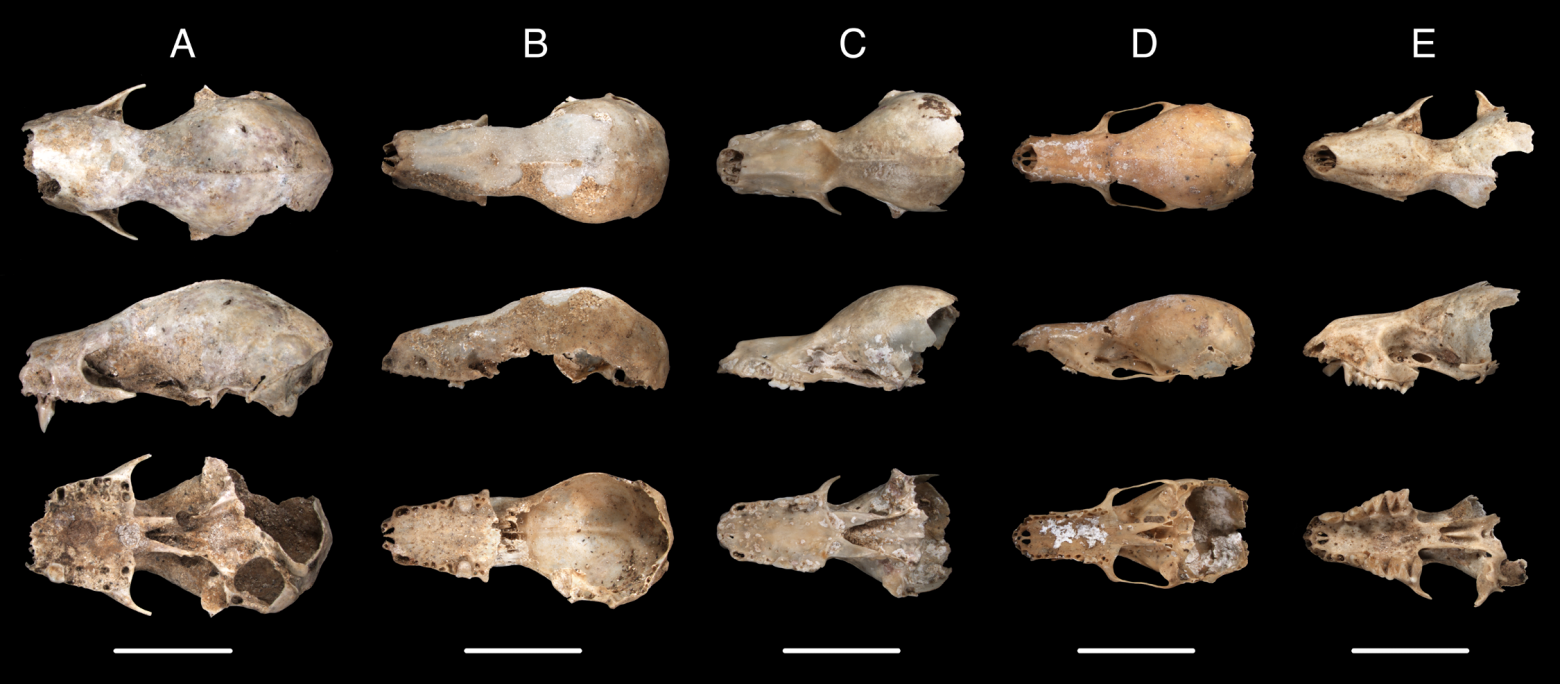
Fossil crania of bats from Trouing Jean Paul, Haiti, in the family Phyllostomidae shown as dorsal (top), lateral (middle), and ventral (bottom) views. Left to right: *Brachyphylla nana* (A; UF 307430), *Phyllonycteris poeyi* (B; UF 307397), *Erophylla bombifrons* (C; UF 282439), *Monophyllus redmani* (D; UF 282169), and *Macrotus waterhousii* (E; UF 282171).

**Fig 5:**
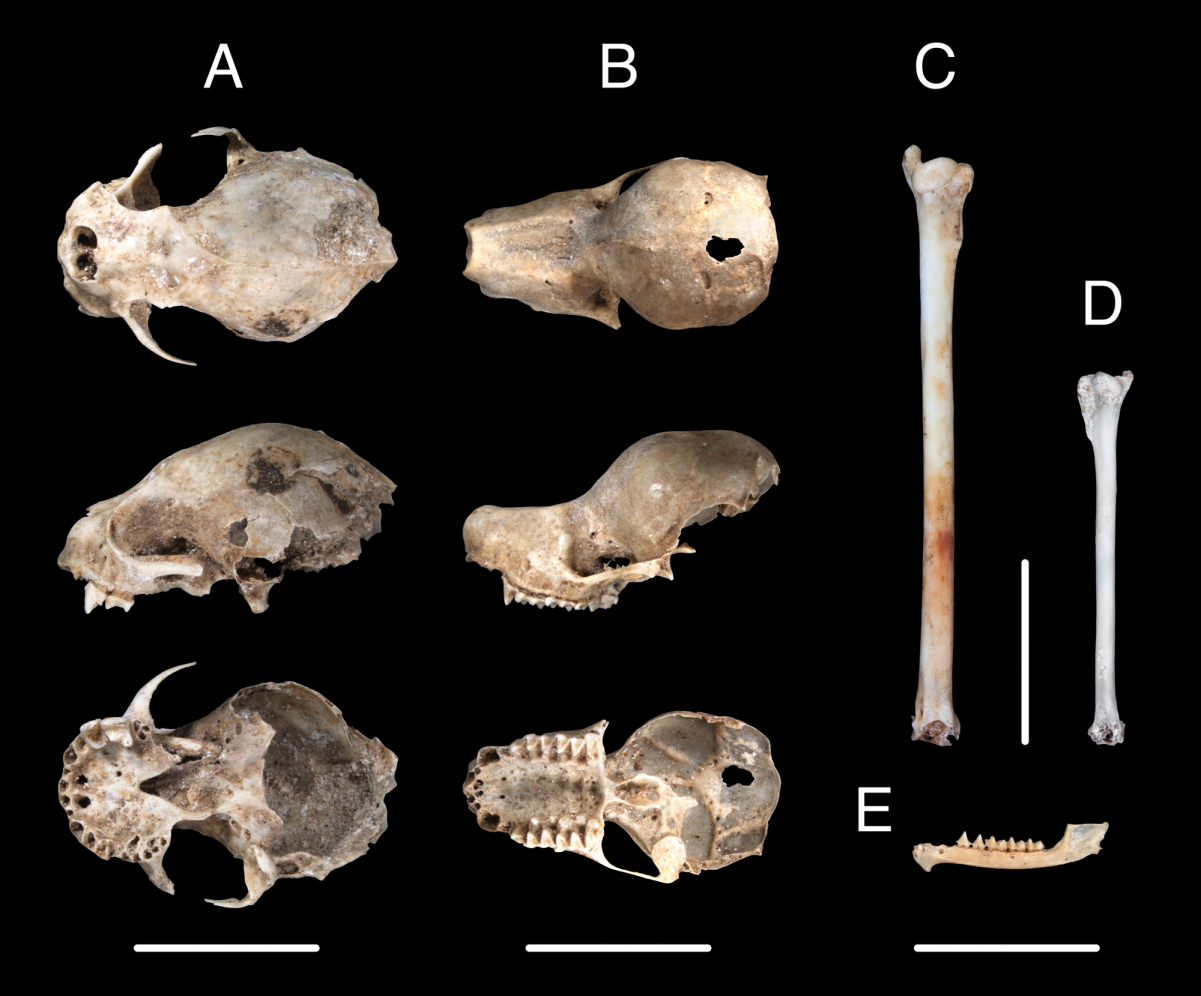
Fossil crania, humeri, and dentary of bats from Trouing Jean Paul, Haiti. Crania shown as dorsal (top), lateral (middle), and ventral (bottom) views. Left to right: Phyllostomidae—*Phyllops falcatus* (A; UF281804), Mormoopidae—*Pteronotus parnellii* (B; UF282717), *Pteronotus quadridens* (D, humerus; UF 297866), Molossidae—*Nyctinomops macrotis* (C, humerus; UF 297865, and Natalidae—*Chilonatalus micropus* (E, dentary; UF 282987).

We identified 15 species of bats in five families from 2940 fossil elements at TJP (Table 2, Figs 3–5). UF catalog numbers for each specimen examined are provided in S2 Table. This bat community represents about 27% and 83% of the diversity of bats in the Caribbean at the level of species and family, respectively. In all, 75% of the extant bat species of Hispaniola and all families except Noctilionidae are represented in in the TJP assemblage. Four abundant species (*Eptesicus fuscus*, *Lasiurus minor*, *Monophyllus redmani*, and *Phyllonycteris poeyi*) make up 94% of the bat fossils identified from TJP. Crania and dentaries were the most prevalent diagnostic elements in the fossil sample, but we also identified femora, humeri, pelves, and radii.

**Table 2:** Fossil bat species identified in this study from Trouing Jean Paul, Parc National La Visite, Massif de la Selle, Haiti.

Family	Scientific name	Number of fossils	MNI	Status[29,30]	Roost preference
Molossidae	*Nyctinomops macrotis*	3	2	E	C, S
	*Tadarida brasiliensis*	47	31	E	C, S
Mormoopidae	*Pteronotus parnellii*	7	2	E	HC
	Pteronotus quadridens^*^	1	1	E	HC
Natalidae	Chilonatalus micropus^*^	3	1	U	HC
	Natalus major^*^	2	2	E	HC
Phyllostomidae	Brachyphylla nana^*^	21	12	E	C
	Erophylla bombifrons^*^	39	21	E	HC
	*Macrotus waterhousii*	21	10	E	C, S
	Monophyllus redmani^*^	277	182	E	HC
	Phyllonycteris poeyi^*^	134	117	E	HC
	Phyllops falcatus^*^	4	3	E	V
Vespertilionidae	*Eptesicus fuscus*	1944	1086	E	C
	*Lasiurus cinereus*	35	22	U	V
	Lasiurus minor^*^	401	280	E	V
Totals	15 species	2940	1772		

Asterisks (*) represent species that are endemic to the Caribbean.

Status of each species in Haiti listed as extant (E) and unknown (U). Roost preference categories listed as cool cave (C), hot cave (HC), man-made structure (S), and vegetation (V). See methods for description of estimates of minimum number of individuals (MNI).

The most abundant species in the TJP fauna is *Eptesicus fuscus* (Big Brown Bat; N = 1944; Fig 3), which has a Caribbean distribution that includes the Greater Antilles and The Bahamas. This aerial insectivore also has a wide continental distribution in North, Central, and northern South America [31]. The second most common species is *Lasiurus minor* (Minor Red Bat; N = 401; Fig 3), a solitary insectivorous species that roosts primarily in trees and is known to occur at high elevations in the Caribbean [30]. The third and fourth most abundant species found in TJP are the Caribbean phyllostomid endemics *Monophyllus redmani* (Greater Antillean Long-tongued Bat; N = 277) and *Phyllonycteris poeyi* (Cuban Flower Bat; N = 134; Fig 4). These primarily nectarivorous bats are distributed throughout the Greater Antilles, are strictly cave-dwelling, and often roost together in the same caves [9]. Both of these species form colonies up to tens of thousands of individuals and are associated with hot caves [9,30]. “Hot caves” are characterized by their high temperatures (28–40˚C) and humidity (>90%) that are believed to be caused mainly by the radiating body heat from large colonies of bats and heat/humidity associated with decomposing guano [32,33].

The remaining 11 fossil bat species from TJP are represented by fewer than 50 elements each and include members of the families Molossidae, Mormoopidae, Natalidae, Phyllostomidae, and Vespertilionidae (Table 2, Figs 3–5). Two of these species are especially noteworthy. First, although commonly included in lists of the bats of Haiti due to its presence in Dominican Republic, we report here the first record of *Chilonatalus micropus* from Haiti identified from three fossil elements (Table 2; Fig 5). Second, *Lasiurus cinereus* has been documented only once in the Caribbean from a single specimen collected in Dominican Republic, which was believed to be a wayward migrant [34]. The presence of 35 fossils of *L*. *cinereus* in TJP suggests that this species is more likely a resident than a migrant (Table 2; Fig 3).

Sixty percent of the bats at TJP (9 of 15) are Caribbean endemics and include extant phytophagous and insectivorous species. Nine of the 15 TJP bat species roost exclusively in caves, of which seven use primarily hot caves (Table 2). In addition to the strict cave-dwelling bats, three of the species found in TJP roost in vegetation, and three of them facultatively use caves and man-made structures (Table 2).

The grid coordinates for 736 of the fossil bat specimens, representing 14 of the 15 species in TJP, were identical to those used by Steadman and Takano [22] for AMS ^14^C dating. These included *Brachyphylla nana* (N = 5), *Chilonatalus micropus* (N = 3), *Eptesicus fuscus* (N = 443), *Erophylla bombifrons* (N = 10), *Lasiurus cinereus* (N = 9), *L*. *minor* (N = 121), *Macrotus waterhousii* (N = 12), *Monophyllus redmani* (N = 85), *Natalus major* (N = 1), *Nyctinomops macrotis* (N = 2), *Phyllonycteris poeyi* (N = 29), *Phyllops falcatus* (N = 1), *Pteronotus parnellii* (N = 2), and *Tadarida brasiliensis* (N = 13). The ^14^C dates from these grid coordinates ranged from 1690–1530 Cal BP to 680–570 Cal BP with 95% confidence (2δ; Table 3), i.e., from about 1600 to 600 years ago.

**Table 3:** Accelerator Mass Spectrometer (AMS) radiocarbon (^14^C) dates obtained from bones in superficial deposits at Trouing Jean Paul, Parc National La Visite, Massif de la Selle, Haiti.

Species	Grid coordinates	Sample number	Conventional ^14^C date (yr BP ± 1δ)	Calibrated age (Cal. yr BP, 2δ)
Bn, Ef, Lm, Mr, Pp, Tb	48.7.59.D	Beta-317709	600 ± 30	680–650, 580–570
Ef, Lm, Mr, Ptp, Tb	53.8.62.C	Beta-317708	760 ± 30	760–690
Cm, Eb, Lc, Mr, Nym, Tb	50.5.60.A	Beta-317710	840 ± 30	930–780
Bn, Eb, Ef, Lc, Lm, Mw, Mr, Nm, Pf, Pp, Tb	52.9.62.C	Beta-317706	1000 ± 30	1060–940
Cm, Eb, Lc, Mr, Tb	50.5.60.A	Beta-317711	1010 ± 30	1060–960
Eb, Ef, Lc, Lm, Mr, Pp, Tb	49.6.60.C	Beta-317707	1580 ± 30	1690–1660, 1630–1530

Dates presented from youngest to oldest. Multiple calibration BP age ranges are due to multiple 2δ intercepts in the calibration curve. Species abbreviations in alphabetical order: *Brachyphylla nana* (Bn), *Chilonatalus micropus* (Cm), *Erophylla bombifrons* (Eb), *Eptesicus fuscus* (Ef), *Lasiurus cinereus* (Lc), *Lasiurus minor* (Lm), *Macrotus waterhousii* (Mw), *Monophyllus redmani* (Mr), *Natalus major* (Nm), *Nyctinomops macrotis* (Nym), *Phyllops falcatus* (Pf), *Phyllonycteris poeyi* (Pp), *Pteronotus parnellii* (Ptp), *Tadarida brasiliensis* (Tb).

## Discussion

The study of islands and their biodiversity has contributed to important ecological and evolutionary theories focusing on dispersal, colonization, extinction, and speciation [35,36]. Given the fast pace of human-induced global change, combining data from both living species and fossils provides important insight into how communities have been shaped across time. As the second largest island of the Caribbean, Hispaniola supports some of the greatest diversity of habitats and highest levels of endemism [37–40]. Coincidently, Haiti has one of the highest levels of habitat loss due to farming and logging, and has lost about 98% of its native forest cover [37]. Understanding the effects of such large-scale human alterations of the environment is critical to predicting the biotic future of the island.

Our study compiled data from 36 localities, described the fossil bat community of Trouing Jean Paul (TJP), Haiti, and provides a thorough review of the living and fossil bat biodiversity of one of the most environmentally imperiled countries in the Neotropics. Previous accounts of Hispaniolan bats documented 18 species in both countries (Dominican Republic and Haiti). Herein we add two recent records, *Lasiurus cinereus* and *Pteronotus macleayii*, which have not been previously included. The prevalence of remains of *L*. *cinereus* in Trouing Jean Paul, Haiti (TJP) shows that this species resides in Hispaniola (Table 2). Based on a single record, *Pteronotus macleayii* is currently known only from Haiti, and together with *L*. *cinereus*, brings the number of bat species in Haiti to 20 (Table 1). Our results, combined with those of previous studies, increase the total number of bats currently occurring on Hispaniola to 20 species.

The fossil bat assemblage of Trouing Jean Paul (TJP) accumulated during an 1100-year interval in the late Holocene (~1690–570 Cal BP). The bat fossils were deposited before the arrival of Europeans and Africans to Hispaniola, which occurred in 1492 AD (which equals 458 Cal BP [41]), although the fossils postdate the colonization of Hispaniola by Amerindians (6–5 ka [42,43]) by about 4 ky. The late Holocene montane bat fauna around TJP was likely sampled by two species of owl, *Tyto alba* and *T*. *glaucops*. While some studies suggest fossil biases in predator-derived sites due to feeding preference of the predator [44,45], we are confident that the bat diversity sampled by these owls is representative of the bat fauna of TJP because all species have been previously documented in the diets of tytonid owls and are well within the range of prey body mass taken by the owls [28]. The foraging radius of *T*. *alba* is up to 5.6 km but usually less [46]; that of the smaller *T*. *glaucops* is unknown but unlikely to be greater than in *T*. *alba*.

The two most common species of bats in the TJP community belong to the family Vespertilionidae and are either found in a few localities or considered uncommon on most Caribbean islands (Table 1). *Eptesicus fuscus* in the Caribbean forms small colonies of a few hundred individuals that are frequently found near the entrance of caves [29,30]. In contrast, *Lasiurus minor* is solitary and roosts among the leaves of trees but apparently never in tree hollows, buildings, or caves [29]. Because of its roosting preference, *L*. *minor* is seldom captured by scientists in the Caribbean, and is represented by fewer than 10 records from Hispaniola in museum collections (www.vertnet.org). Both *Eptesicus fuscus* and *Lasiurus minor* have a swift flight pattern with low maneuverability and typically forage in open areas [29]. Ashy-faced owls (*Tyto glaucops*) and barn owls (*T*. *alba*) routinely roost in caves and sinkholes where they can capture bats in flight, and they are also known prey upon bat species that do not live in caves [28,30]. It is therefore likely that the roosting and foraging habitat preferences of *Eptesicus fuscus* and *Lasiurus minor* render them easy prey to aerial predators such as owls. The TJP sample of *L*. *minor* is the largest documented so far for the Caribbean. Given the high incidence of *Eptesicus fuscus* fossils found at TJP, it is plausible that a colony of this bat was present within TJP.

*Chilonatalus micropus* in TJP represents the first record of this species from Haiti. This bat exclusively roosts in small colonies in hot caves and typically forages in densely cluttered mesic environments [47]. In Dominican Republic, *C*. *micropus* is considered rare and has been documented in two northeast localities in Samaná Province and two southwest localities in Barahona Province. Both *Tyto alba* and *T*. *glaucops* frequently prey upon very small vertebrates such as the 0.6 g Tuck-weep Frog (*Eleutherodactylus abbotti* [28]), suggesting that they could effectively capture *Chilonatalus micropus*, which weighs only 2.6 g and has a characteristic slow flight pattern [47,48]. The relatively low numbers of *C*. *micropus* in TJP may result from its preference for roosting in hot caves, and because this species typically forages in densely forested areas where it is less likely to be captured by the owls.

The presence of *Lasiurus cinereus* in TJP stands out among this diverse bat fauna. This bat is solitary, insectivorous, and roosts primarily among the foliage in trees [34]. It is one of the most widespread of American bats and occurs from Canada (North America) to Brazil, northern Argentina, and Chile (South America; [34]). *Lasiurus cinereus* also occurs in the Galápagos Islands (Ecuador) and is the only bat that has colonized Hawai’i, which is one of the longest dispersal distances known to have been traversed by bats [49]. *Lasiurus cinereus* has been reported from Bermuda (3 specimens) and Hispaniola (1 specimen) but, until now, these individuals were considered migrants [34]. The verbatim locality for the single Hispaniolan specimen (USNM 105704) is “Dominican Republic” and lacks specific details to accurately determine its provenance. The estimated minimum number of individuals of *L*. *cinereus* (MNI = 22) in the TJP sample is comparable to some colonial cave-dwelling species that are considered common in the Caribbean (e.g., *Tadarida brasiliensis*, MNI = 31; *Brachyphylla nana*, MNI = 12; *Erophylla bombifrons*, MNI = 21; Table 2). Although only a single living specimen has been collected, the relatively young radiocarbon dates of *L*. *cinereus* samples (~930–780 Cal BP; Table 3) at TJP suggest that resident (non-migratory) populations of this species are likely present. The paucity of living records of *L*. *cinereus* in the Caribbean probably reflects the difficulty of catching these bats in mist nets and lack of adequate biodiversity inventory efforts in Hispaniola.

Both *Lasiurus minor* and *L*. *cinereus* were found commonly in TJP, yet they are considered rare in the Caribbean. TJP lies at about 1800 m elevation within a 3000 ha area of pine forest, broadleaf cloud forest, savanna, and juniper forest in Massif de la Selle [21,50]. Substantial areas to the east of Massif de la Selle (e.g., Calalo and Savane Bourrique in Haiti, and Pedernales and Bahoruco in Dominican Republic) are also characterized by mountain ranges over 1500 m. While Caribbean records of *L*. *minor* do not necessarily represent high elevation localities, the majority are from topographically heterogeneous forested habitats with steep elevational gradients that drastically increase in elevation. The single *L*. *cinereus* specimen from Dominican Republic lacks specific locality information. Nonetheless, the occurrence of *L*. *cinereus* in the TJP deposits suggests that it inhabits high elevation forests in Haiti and, given the size of the foraging range of the owls, this species was likely part of the resident bats of the area. It therefore seems likely that the mountain ranges of southeastern Haiti and southwestern Dominican Republic contain adequate habitat to sustain populations of *L*. *minor* and *L*. *cinereus* that have eluded modern scientific surveys but are efficiently sampled by owls.

The long-term persistence of most species of bats in Haiti (and in Hispaniola in general) stands in sharp contrast to the situation with non-volant mammals, about 80% of which have become extinct during the late Quaternary [2,3]. Two likely factors in the greater resilience of bats may be their roosting habits in caves (which are less subjected to human impact than non-cave habitats) and their lack of appeal to humans as food. The considerable late Holocene extirpation of bats found on the Bahamian island of Abaco [8], however, demonstrates that even bat communities can be highly vulnerable to loss on relatively small islands. The large size and high relief of Hispaniola has apparently buffered its bat communities from rapid declines. However, persistence of high-elevation forests may have been key in the preservation of the bat fauna. Ongoing deforestation, if it encompasses higher elevation areas, may have a rapid and devastating effect on the bats of Hispaniola.

## Supporting information

S1 Table Descriptions for all localities where living or fossil bats have been documented in Haiti. Verbatim elevation (in meters) provided for previously published localities if available. Elevation for new localities reported in this study estimated via GoogleEarth.
(XLSX)

S2 Table List of specimens examined per species for Trouing Jean Paul, Haiti, from the UF Vertebrate Paleontology collection in Gainesville, FL. (PDF)
