# Supplementary material for: The bat community of Haiti and evidence for its long-term persistence at high elevations
Source: PLoS One. 2017 Jun 2;12(6):e0178066. doi: 10.1371/journal.pone.0178066 (PMC5456054; doi:10.1371/journal.pone.0178066)
Supplement: S2 Table — (PDF) [file pone.0178066.s002.pdf]

S2 Table. List of specimens examined per species for Trouing Jean Paul, Haiti, from the UF Vertebrate Paleontology collection in Gainesville, FL.

| Species | UF catalog numbers |
|---|---|
| *Nyctinomops macrotis* | 297865 |
| *Tadarida brasiliensis* | 281528, 281650, 281707, 281739, 281912, 281913, 281914, 281978, 282370, 282400, 282411, 282413, 282491, 282589, 282603, 282622, 282623, 282706, 282714, 282715, 282716, 282749, 282791, 282817, 282973, 282988, 282989, 282990, 282993, 282994, 282998, 282999, 307337, 307419, 307467, 307468, 307469, 307474, 307475, 307476, 308414, 308928 |
| *Pteronotus parnellii* | 281579, 282179, 282717, 282811, 282992 |
| *Pteronotus quadridens* | 297866 |
| *Chilonatalus micropus* | 282987 |
| *Natalus major* | 281932, 307471 |
| *Brachyphylla nana* | 281620, 281784, 281899, 281910, 281942, 282186, 282297, 282299, 282455, 282635, 282655, 282718, 282894, 282918, 282922, 307430, 307470, 307473, 308458 |
| *Erophylla bombifrons* | 281500, 281542, 281566, 281924, 281993, 282276, 282311, 282439, 282443, 282532, 282547, 282656, 282699, 282707, 282708, 282755, 282777, 282891, 282966, 307310, 307331, 307364, 307369, 307538, 307777, 307873, 307947, 308013, 308014, 308036, 308037, 308065, 308066, 308449, 308497 |
| *Macrotus waterhousii* | 282171, 307265 |
| *Monophyllus redmani* | 281521, 281537, 281545, 281560, 281578, 281606, 281608, 281619, 281627, 281636, 281662, 281677, 281678, 281679, 281689, 281706, 281710, 281714, 281781, 281786, 281788, 281799, 281802, 281812, 281819, 281825, 281826, 281841, 281868, 281871, 281873, 281889, 281896, 281898, 281928, 281938, 281965, 281977, 281982, 281994, 281995, 281996, 281999, 282153, 282155, 282157, 282162, 282165, 282169, 282172, 282173, 282174, 282175, 282184, 282211, 282213, 282226, 282234, 282252, 282266, 282274, 282275, 282282, 282283, 282304, 282306, 282310, 282312, 282329, 282331, 282334, 282343, 282344, 282375, 282381, 282385, 282393, 282401, 282431, 282433, 282437, 282446, 282447, 282461, 282464, 282465, 282469, 282477, 282487, 282496, 282499, 282503, 282513, 282529, 282544, 282548, 282559, 282583, 282585, 282594, 282595, 282641, 282651, 282653, 282681, 282702, 282704, 282709, 282742, 282751, 282756, 282767, 282783, 282784, 282792, 282801, 282826, 282849, 282889, 282890, 282892, 282893, 282946, 282959, 282965, 282969, 307227, 307228, 307259, 307261, 307269, 307290, 307296, 307298, 307316, 307327, 307333, 307338, 307363, 307384, 307388, 307416, 307423, 307436, 307441, 307472, 307481, 307509, 307510, 307518, 307519, 307520, 307521, 307586, 307587, 307588, 307589, 307646, 307647, 307648, 307649, 307650, 307687, 307688, 307689, 307787, 307788, 307789, 307790, 307791, 307792, 307809, 307855, 307856, 307857, 307858, 307859, 307860, 307861, 307862, 307863, 307864, 307865, 307866, |

| | |
|---|---|
| *Monophyllus redmani* | 307867, 307868, 307869, 307870, 307871, 307872, 307919, 307948, 308041, 308042, 308043, 308044, 308045, 308063, 308064, 308327, 308328, 308329, 308330, 308331, 308332, 308431, 308432, 308433, 308434, 308435, 308436, 308437, 308438, 308439, 308440, 308441, 308442, 308443, 308444, 308445, 308446, 308447, 308448, 308499, 308500, 308992, 308993, 308994, 308995, 308996, 308997 |
| *Phyllonycteris poeyi* | 281544, 281554, 281569, 281570, 281576, 281577, 281584, 281609, 281618, 281635, 281651, 281681, 281770, 281810, 281815, 281851, 281902, 281957, 281964, 281969, 281973, 281992, 281997, 281998, 282152, 282156, 282158, 282185, 282190, 282207, 282235, 282269, 282303, 282325, 282332, 282371, 282376, 282377, 282391, 282397, 282432, 282438, 282440, 282444, 282450, 282460, 282542, 282543, 282554, 282555, 282564, 282592, 282646, 282857, 282883, 282939, 282940, 282948, 282953, 282957, 283000, 307319, 307325, 307339, 307346, 307357, 307376, 307397, 307410, 307415, 307477, 307508, 307517, 307539, 307540, 307541, 307542, 307543, 307544, 307582, 307583, 307584, 307585, 307641, 307642, 307643, 307644, 307645, 307690, 307691, 307692, 307693, 307694, 307778, 307779, 307780, 307781, 307782, 307783, 307784, 307785, 307786, 307810, 307874, 307875, 307876, 307877, 307878, 307879, 307920, 307922, 308015, 308016, 308017, 308038, 308039, 308040, 308333, 308334, 308335, 308336, 308450, 308451, 308452, 308453, 308454, 308455, 308456, 308457, 308493, 308494, 308495, 308496, 308498 |
| *Phyllops falcatus* | 281610, 281804, 282991, 307618 |
| *Eptesicus fuscus* | 281506, 281509, 281510, 281511, 281512, 281514, 281516, 281517, 281518, 281519, 281520, 281522, 281523, 281524, 281525, 281530, 281531, 281533, 281535, 281536, 281540, 281541, 281543, 281547, 281549, 281550, 281551, 281552, 281553, 281555, 281556, 281558, 281559, 281561, 281562, 281563, 281564, 281565, 281568, 281571, 281572, 281573, 281574, 281575, 281580, 281581, 281583, 281585, 281587, 281588, 281589, 281590, 281591, 281592, 281593, 281594, 281595, 281597, 281598, 281599, 281600, 281602, 281603, 281604, 281605, 281607, 281613, 281614, 281615, 281617, 281621, 281623, 281625, 281626, 281628, 281630, 281631, 281632, 281633, 281634, 281637, 281638, 281639, 281640, 281641, 281642, 281643, 281644, 281645, 281646, 281647, 281648, 281649, 281652, 281653, 281655, 281656, 281658, 281660, 281664, 281665, 281666, 281667, 281669, 281670, 281671, 281672, 281673, 281674, 281675, 281676, 281680, 281683, 281685, 281686, 281690, 281692, 281693, 281694, 281695, 281696, 281697, 281698, 281699, 281700, 281702, 281704, 281709, 281711, 281713, 281715, 281716, 281717, 281718, 281719, 281720, 281721, 281722, 281723, 281726, 281728, 281729, 281730, 281731, 281732, 281733, 281735, 281736, 281737, 281738, 281740, 281741, 281742, 281743, 281744, 281745, 281747, 281748, 281750, 281751, 281752, 281754, 281755, 281756, 281757, 281760, 281761, 281762, 281764, 281765, 281766, 281768, 281769, 281771, 281772, 281773, 281774, 281775, 281776, 281777, 281778, 281779, 281780, 281782, 281783, 281785, 281787, 281790, 281791, 281792, 281793, 281794, |

| | |
|---|---|
| *Eptesicus fuscus* | 281796, 281797, 281798, 281800, 281801, 281805, 281806, 281807, 281808, 281811, 281813, 281814, 281816, 281817, 281818, 281820, 281821, 281822, 281823, 281824, 281827, 281828, 281829, 281831, 281832, 281833, 281837, 281838, 281839, 281840, 281842, 281843, 281844, 281846, 281847, 281848, 281854, 281855, 281856, 281859, 281860, 281861, 281862, 281863, 281865, 281866, 281867, 281870, 281872, 281874, 281876, 281877, 281878, 281879, 281880, 281882, 281883, 281884, 281885, 281886, 281887, 281888, 281890, 281891, 281893, 281894, 281895, 281897, 281900, 281901, 281904, 281905, 281906, 281907, 281908, 281909, 281911, 281915, 281917, 281921, 281922, 281929, 281930, 281931, 281933, 281935, 281936, 281937, 281939, 281940, 281941, 281943, 281944, 281946, 281947, 281949, 281950, 281951, 281952, 281953, 281954, 281955, 281956, 281959, 281960, 281962, 281963, 281966, 281967, 281968, 281971, 281972, 281974, 281976, 281979, 281980, 281981, 281983, 281985, 281986, 281987, 281988, 281989, 282154, 282159, 282161, 282163, 282164, 282167, 282168, 282170, 282176, 282177, 282178, 282180, 282181, 282182, 282187, 282188, 282191, 282192, 282193, 282196, 282197, 282198, 282199, 282200, 282201, 282202, 282203, 282204, 282206, 282208, 282209, 282210, 282212, 282215, 282216, 282221, 282222, 282223, 282224, 282225, 282227, 282228, 282229, 282230, 282231, 282232, 282233, 282236, 282237, 282238, 282239, 282240, 282242, 282243, 282244, 282246, 282247, 282248, 282250, 282251, 282253, 282254, 282255, 282256, 282257, 282258, 282259, 282260, 282262, 282263, 282265, 282268, 282270, 282271, 282272, 282273, 282277, 282279, 282280, 282281, 282284, 282285, 282286, 282287, 282288, 282289, 282290, 282291, 282292, 282294, 282296, 282298, 282300, 282301, 282302, 282307, 282308, 282313, 282314, 282315, 282316, 282317, 282318, 282319, 282320, 282321, 282322, 282323, 282324, 282326, 282327, 282328, 282333, 282335, 282336, 282337, 282338, 282339, 282340, 282346, 282347, 282348, 282349, 282350, 282351, 282352, 282353, 282354, 282355, 282356, 282357, 282358, 282359, 282360, 282361, 282363, 282364, 282365, 282366, 282369, 282372, 282373, 282374, 282378, 282379, 282380, 282382, 282386, 282387, 282388, 282389, 282390, 282392, 282394, 282395, 282396, 282398, 282399, 282402, 282403, 282404, 282405, 282406, 282407, 282408, 282409, 282410, 282414, 282415, 282416, 282417, 282426, 282428, 282429, 282434, 282435, 282436, 282441, 282442, 282445, 282448, 282449, 282451, 282452, 282453, 282454, 282456, 282457, 282458, 282459, 282462, 282463, 282467, 282468, 282472, 282473, 282474, 282475, 282478, 282479, 282480, 282481, 282482, 282483, 282484, 282485, 282486, 282488, 282489, 282492, 282493, 282495, 282497, 282500, 282502, 282504, 282505, 282506, 282507, 282508, 282509, 282510, 282511, 282514, 282515, 282516, 282517, 282518, 282519, 282520, 282521, 282522, 282523, 282525, 282526, 282527, 282528, 282530, 282531, 282533, 282535, 282536, 282537, 282538, 282540, 282541, 282545, 282546, 282549, 282550, 282551, 282552, 282553, 282556, 282557, 282558, 282561, 282562, 282563, 282586, 282587, |

| | |
|---|---|
| *Eptesicus fuscus* | 282588, 282590, 282593, 282596, 282597, 282598, 282599, 282601, 282602, 282604, 282606, 282607, 282608, 282611, 282612, 282613, 282614, 282615, 282617, 282618, 282620, 282624, 282625, 282627, 282628, 282629, 282630, 282631, 282632, 282633, 282634, 282636, 282637, 282638, 282639, 282640, 282642, 282643, 282645, 282647, 282648, 282649, 282654, 282657, 282658, 282659, 282660, 282661, 282662, 282664, 282665, 282666, 282667, 282668, 282669, 282670, 282671, 282672, 282673, 282674, 282675, 282676, 282677, 282678, 282679, 282680, 282682, 282683, 282684, 282685, 282687, 282688, 282689, 282690, 282691, 282692, 282694, 282695, 282696, 282697, 282698, 282700, 282701, 282703, 282705, 282719, 282720, 282721, 282722, 282723, 282724, 282725, 282726, 282727, 282728, 282729, 282730, 282731, 282732, 282733, 282734, 282735, 282736, 282737, 282738, 282739, 282740, 282741, 282744, 282745, 282746, 282747, 282748, 282750, 282752, 282753, 282754, 282757, 282759, 282760, 282762, 282763, 282765, 282766, 282768, 282769, 282770, 282771, 282772, 282774, 282775, 282776, 282778, 282779, 282780, 282781, 282782, 282787, 282788, 282789, 282790, 282794, 282795, 282796, 282797, 282798, 282799, 282800, 282802, 282803, 282804, 282806, 282808, 282809, 282810, 282812, 282813, 282814, 282815, 282819, 282821, 282822, 282823, 282824, 282825, 282827, 282828, 282830, 282831, 282833, 282835, 282836, 282837, 282839, 282840, 282841, 282843, 282844, 282845, 282847, 282848, 282850, 282852, 282853, 282854, 282855, 282856, 282858, 282859, 282861, 282864, 282866, 282867, 282869, 282870, 282871, 282872, 282874, 282876, 282877, 282878, 282880, 282881, 282884, 282885, 282886, 282887, 282895, 282896, 282897, 282898, 282899, 282900, 282901, 282902, 282903, 282904, 282905, 282906, 282907, 282908, 282910, 282912, 282913, 282914, 282915, 282917, 282919, 282920, 282921, 282924, 282925, 282926, 282927, 282930, 282931, 282933, 282934, 282935, 282936, 282937, 282938, 282941, 282942, 282943, 282944, 282947, 282949, 282950, 282951, 282952, 282956, 282958, 282960, 282961, 282962, 282963, 282964, 282967, 282968, 282970, 282971, 282972, 282975, 282976, 282977, 282979, 282980, 282981, 282982, 282984, 282986, 307201, 307203, 307204, 307205, 307207, 307208, 307209, 307210, 307211, 307212, 307213, 307215, 307216, 307217, 307218, 307219, 307220, 307221, 307222, 307223, 307224, 307225, 307226, 307229, 307230, 307231, 307232, 307233, 307234, 307235, 307236, 307237, 307238, 307239, 307240, 307241, 307242, 307244, 307245, 307246, 307248, 307249, 307250, 307251, 307252, 307253, 307255, 307258, 307260, 307263, 307266, 307267, 307268, 307270, 307271, 307272, 307273, 307276, 307277, 307278, 307279, 307280, 307281, 307282, 307283, 307284, 307285, 307286, 307287, 307288, 307289, 307292, 307293, 307294, 307295, 307297, 307299, 307300, 307301, 307302, 307303, 307305, 307306, 307307, 307308, 307309, 307311, 307312, 307313, 307314, 307315, 307317, 307320, 307323, 307324, 307326, 307328, 307329, 307330, 307332, 307334, 307335, 307340, 307342, 307343, 307344, 307345, 307347, 307348, 307349, 307350, 307351, |

| | |
|---|---|
| *Eptesicus fuscus* | 307352, 307353, 307354, 307355, 307356, 307358, 307359, 307360,<br>307362, 307365, 307366, 307367, 307368, 307370, 307372, 307373,<br>307374, 307375, 307377, 307378, 307379, 307380, 307381, 307382,<br>307383, 307385, 307387, 307389, 307390, 307391, 307392, 307393,<br>307394, 307396, 307398, 307399, 307404, 307405, 307406, 307407,<br>307408, 307409, 307411, 307412, 307417, 307418, 307421, 307422,<br>307425, 307428, 307429, 307431, 307433, 307435, 307440, 307442,<br>307443, 307444, 307445, 307446, 307447, 307448, 307449, 307450,<br>307452, 307454, 307455, 307457, 307458, 307459, 307460, 307461,<br>307462, 307464, 307465, 307482, 307483, 307484, 307485, 307486,<br>307487, 307488, 307489, 307490, 307491, 307492, 307493, 307494,<br>307495, 307496, 307497, 307498, 307499, 307500, 307501, 307502,<br>307503, 307504, 307505, 307506, 307507, 307522, 307523, 307524,<br>307525, 307526, 307527, 307528, 307529, 307530, 307531, 307532,<br>307533, 307534, 307535, 307536, 307537, 307549, 307550, 307551,<br>307552, 307553, 307554, 307555, 307556, 307557, 307558, 307559,<br>307560, 307561, 307562, 307563, 307564, 307565, 307566, 307567,<br>307568, 307569, 307570, 307571, 307572, 307573, 307574, 307575,<br>307576, 307577, 307578, 307579, 307590, 307591, 307592, 307593,<br>307594, 307595, 307596, 307597, 307598, 307599, 307600, 307601,<br>307603, 307604, 307605, 307607, 307609, 307610, 307612, 307613,<br>307614, 307615, 307616, 307619, 307620, 307621, 307622, 307623,<br>307624, 307625, 307626, 307627, 307628, 307629, 307630, 307631,<br>307632, 307633, 307634, 307635, 307636, 307637, 307638, 307639,<br>307640, 307657, 307658, 307659, 307660, 307661, 307662, 307663,<br>307664, 307665, 307666, 307667, 307668, 307669, 307670, 307671,<br>307672, 307673, 307674, 307675, 307676, 307677, 307678, 307679,<br>307680, 307681, 307682, 307683, 307684, 307685, 307686, 307698,<br>307699, 307700, 307701, 307702, 307703, 307704, 307705, 307706,<br>307707, 307708, 307709, 307710, 307711, 307712, 307713, 307714,<br>307715, 307716, 307717, 307718, 307719, 307720, 307721, 307722,<br>307723, 307724, 307725, 307726, 307727, 307728, 307729, 307730,<br>307731, 307732, 307733, 307734, 307735, 307736, 307737, 307738,<br>307739, 307740, 307741, 307742, 307743, 307744, 307745, 307746,<br>307747, 307748, 307749, 307750, 307751, 307752, 307753, 307754,<br>307755, 307756, 307757, 307758, 307759, 307760, 307761, 307762,<br>307763, 307764, 307765, 307766, 307767, 307768, 307769, 307770,<br>307771, 307772, 307773, 307774, 307775, 307776, 307811, 307812,<br>307813, 307814, 307815, 307816, 307817, 307818, 307819, 307820,<br>307821, 307822, 307823, 307824, 307825, 307826, 307827, 307828,<br>307829, 307830, 307831, 307832, 307833, 307834, 307835, 307836,<br>307837, 307838, 307839, 307840, 307841, 307842, 307843, 307844,<br>307845, 307846, 307847, 307848, 307849, 307850, 307851, 307852,<br>307853, 307854, 307921, 307923, 307924, 307925, 307926, 307927,<br>307928, 307929, 307930, 307931, 307932, 307933, 307934, 307935,<br>307936, 307937, 307938, 307939, 307940, 307941, 307942, 307943,<br>307944, 307945, 307946, 307951, 307952, 307953, 307954, 307955,<br>307956, 307957, 307958, 307959, 307960, 307961, 307962, 307963, |

| | |
|---|---|
| *Eptesicus fuscus* | 307964, 307965, 307966, 307967, 307968, 307969, 307970, 307971, 307972, 307973, 307974, 307975, 307976, 307977, 307978, 307979, 307980, 307981, 307982, 307983, 307984, 307985, 307986, 307987, 307988, 307989, 307990, 307991, 307992, 307993, 307994, 307995, 307996, 307997, 307998, 307999, 308000, 308001, 308002, 308003, 308004, 308005, 308006, 308007, 308008, 308009, 308010, 308011, 308012, 308067, 308068, 308069, 308070, 308071, 308072, 308073, 308074, 308075, 308076, 308077, 308078, 308079, 308080, 308081, 308082, 308083, 308084, 308085, 308086, 308087, 308088, 308089, 308090, 308091, 308092, 308093, 308094, 308095, 308096, 308097, 308098, 308099, 308100, 308101, 308102, 308103, 308104, 308105, 308106, 308107, 308108, 308109, 308110, 308111, 308112, 308113, 308114, 308115, 308116, 308117, 308118, 308119, 308120, 308121, 308122, 308123, 308124, 308125, 308126, 308127, 308128, 308129, 308130, 308131, 308132, 308133, 308134, 308135, 308136, 308137, 308138, 308139, 308140, 308141, 308142, 308143, 308144, 308145, 308146, 308147, 308148, 308149, 308150, 308151, 308152, 308153, 308154, 308155, 308156, 308157, 308158, 308159, 308160, 308162, 308163, 308164, 308165, 308166, 308167, 308168, 308169, 308170, 308171, 308172, 308173, 308174, 308175, 308176, 308177, 308178, 308179, 308180, 308181, 308182, 308183, 308184, 308185, 308186, 308187, 308188, 308189, 308190, 308191, 308192, 308193, 308194, 308195, 308196, 308197, 308198, 308199, 308200, 308201, 308202, 308203, 308204, 308205, 308206, 308207, 308208, 308209, 308210, 308211, 308212, 308213, 308214, 308215, 308216, 308217, 308218, 308219, 308220, 308221, 308222, 308223, 308224, 308225, 308226, 308227, 308228, 308229, 308230, 308231, 308232, 308233, 308234, 308235, 308236, 308237, 308238, 308239, 308240, 308241, 308242, 308243, 308244, 308245, 308246, 308247, 308248, 308249, 308250, 308251, 308252, 308253, 308254, 308255, 308256, 308257, 308258, 308259, 308260, 308261, 308262, 308263, 308264, 308265, 308266, 308267, 308268, 308269, 308283, 308284, 308285, 308286, 308287, 308288, 308289, 308290, 308291, 308292, 308293, 308294, 308295, 308296, 308297, 308298, 308299, 308300, 308301, 308302, 308303, 308304, 308305, 308306, 308307, 308308, 308309, 308310, 308311, 308312, 308313, 308314, 308315, 308316, 308317, 308318, 308319, 308320, 308321, 308322, 308323, 308324, 308325, 308326, 308338, 308339, 308340, 308341, 308342, 308343, 308344, 308345, 308346, 308347, 308348, 308349, 308350, 308351, 308352, 308353, 308354, 308355, 308356, 308357, 308358, 308359, 308360, 308361, 308362, 308363, 308364, 308365, 308366, 308367, 308368, 308369, 308370, 308371, 308372, 308373, 308373, 308374, 308375, 308376, 308377, 308378, 308379, 308380, 308381, 308382, 308383, 308384, 308385, 308386, 308387, 308388, 308389, 308390, 308391, 308392, 308393, 308394, 308395, 308396, 308397, 308398, 308399, 308400, 308401, 308402, 308403, 308404, 308405, 308406, 308407, 308408, 308409, 308410, 308411, 308412, 308413, 308459, 308460, 308461, 308462, 308463, 308464, 308465, 308466, 308467, 308468, 308469, 308470, |

| | |
|---|---|
| *Eptesicus fuscus* | 308471, 308472, 308473, 308474, 308475, 308476, 308477, 308478, 308479, 308480, 308481, 308482, 308483, 308484, 308897, 308898, 308899, 308900, 308901, 308902, 308907, 308908, 308909, 308919, 308922, 308923, 308924, 308925, 308926, 308927, 308929, 308934, 308938, 308939, 308940, 308941, 308942, 308943, 308944, 308945, 308946, 308947, 308948, 308949, 308950, 308951, 308952, 308953, 308955, 308956, 308957, 308958, 308959, 308960, 308961, 308962, 308976, 308977, 308978, 308979, 308980, 308981, 308982, 308983, 308984, 308985, 308986, 308987, 308988, 308989, 308990, 308991, 309500, 309501, 309502, 309503, 309504, 309505, 309507, 309508, 309509, 309510, 309511, 309512, 309519, 309520 |
| *Lasiurus cinereus* | 281539, 281567, 281616, 281622, 281654, 281663, 281668, 281688, 281712, 281724, 281727, 281758, 281875, 281916, 281926, 281975, 282166, 282368, 282383, 282591, 282605, 282710, 282711, 282712, 282713, 282761, 282773, 282834, 282851, 282995, 282996, 282997, 307321, 307463, 307466 |
| *Lasiurus minor* | 281501, 281502, 281503, 281504, 281505, 281507, 281508, 281513, 281515, 281526, 281527, 281529, 281532, 281534, 281538, 281546, 281557, 281601, 281624, 281659, 281661, 281682, 281684, 281687, 281691, 281701, 281703, 281705, 281708, 281734, 281767, 281789, 281795, 281803, 281809, 281830, 281834, 281835, 281836, 281845, 281849, 281850, 281852, 281853, 281857, 281858, 281864, 281869, 281881, 281892, 281903, 281919, 281920, 281923, 281925, 281927, 281934, 281945, 281948, 281958, 281961, 281970, 281984, 281990, 281991, 282183, 282189, 282194, 282195, 282205, 282214, 282217, 282218, 282219, 282220, 282245, 282249, 282261, 282264, 282267, 282293, 282309, 282330, 282341, 282342, 282345, 282362, 282367, 282384, 282412, 282427, 282466, 282470, 282471, 282476, 282490, 282494, 282498, 282501, 282512, 282539, 282560, 282584, 282600, 282609, 282610, 282616, 282619, 282621, 282626, 282650, 282652, 282686, 282693, 282743, 282785, 282786, 282805, 282807, 282816, 282818, 282829, 282832, 282838, 282842, 282846, 282860, 282862, 282863, 282865, 282868, 282873, 282875, 282879, 282882, 282909, 282911, 282929, 282932, 282945, 282954, 282955, 282974, 282978, 282983, 282985, 307243, 307254, 307256, 307257, 307262, 307291, 307304, 307318, 307336, 307341, 307361, 307371, 307395, 307400, 307401, 307402, 307403, 307413, 307414, 307420, 307424, 307426, 307427, 307432, 307434, 307437, 307438, 307439, 307451, 307453, 307456, 307478, 307479, 307480, 307511, 307512, 307513, 307514, 307515, 307516, 307545, 307546, 307547, 307548, 307580, 307581, 307602, 307606, 307608, 307611, 307617, 307651, 307652, 307653, 307654, 307655, 307656, 307695, 307696, 307697, 307793, 307794, 307795, 307796, 307797, 307798, 307799, 307800, 307801, 307802, 307803, 307804, 307805, 307806, 307807, 307808, 307880, 307881, 307882, 307883, 307884, 307885, 307886, 307887, 307888, 307889, 307890, 307891, 307892, 307893, 307894, 307895, 307896, 307897, 307898, 307899, 307900, 307901, 307902, 307903, 307904, 307905, 307906, 307907, 307908, 307909, 307910, 307911, 307912, 307913, |

| | |
|---|---|
| *Lasiurus minor* | 307914, 307915, 307916, 307917, 307918, 307949, 307950, 308018, 308019, 308020, 308021, 308022, 308023, 308024, 308025, 308026, 308027, 308028, 308029, 308030, 308031, 308032, 308033, 308034, 308035, 308046, 308047, 308048, 308049, 308050, 308051, 308052, 308053, 308054, 308055, 308056, 308057, 308058, 308059, 308060, 308061, 308062, 308270, 308271, 308272, 308273, 308274, 308275, 308276, 308277, 308278, 308279, 308280, 308281, 308282, 308415, 308416, 308417, 308418, 308419, 308420, 308421, 308422, 308423, 308424, 308425, 308426, 308427, 308428, 308429, 308430, 308485, 308486, 308487, 308488, 308489, 308490, 308491, 308492, 308492 |
